# Development and validation of machine learning-based diagnostic models using blood transcriptomics for early childhood diabetes prediction

**DOI:** 10.3389/fmed.2025.1636214

**Published:** 2025-07-16

**Authors:** Xin Huang, Di Ouyang, Weiming Xie, Huawei Zhuang, Siyu Gao, Pan Liu, Lizhong Guo

**Affiliations:** ^1^The First Clinical Medical College, Nanjing University of Chinese Medicine, Nanjing, China; ^2^Yulin Hospital of Traditional Chinese Medicine, Yulin, China; ^3^Traditional Chinese Medicine Hospital of Yulin, Yulin, China; ^4^Academic Affairs and Research Management Office, Yulin Campus of Guangxi Medical University, Yulin, Guangxi, China; ^5^Huai’an No.3 People’s Hospital, Huai’an Second Clinical College of Xuzhou Medical University, Huai’an, China

**Keywords:** childhood diabetes, peripheral blood, transcriptomic analysis, machine learning, pediatric biomarkers

## Abstract

**Background:**

Early identification of Type 1 Diabetes Mellitus (T1DM) in pediatric populations is crucial for implementing timely interventions and improving long-term outcomes. Peripheral blood transcriptomic analysis provides a minimally invasive approach for identifying predictive biomarkers prior to clinical manifestation. This study aimed to develop and validate machine learning algorithms utilizing transcriptomic signatures to predict T1DM onset in children up to 46 months before clinical diagnosis.

**Methods:**

We analyzed 247 peripheral blood RNA-sequencing samples from pre-diabetic children and age-matched healthy controls. Differential gene expression analysis was performed using established bioinformatics pipelines to identify significantly dysregulated transcripts. Five feature selection methods (Lasso, Elastic Net, Random Forest, Support Vector Machine, and Gradient Boosting Machine) were employed to optimize gene sets. Nine machine learning algorithms (Decision Tree, Gradient Boosting Machine, K-Nearest Neighbors, Linear Discriminant Analysis, Logistic Regression, Multilayer Perceptron, Naive Bayes, Random Forest, and Support Vector Machine) were combined with selected features, generating 45 unique model combinations. Performance was evaluated using accuracy, precision, recall, and F1-score metrics. Model validation was conducted using quantitative polymerase chain reaction (qPCR) in an independent cohort of six children (three healthy, three diabetic).

**Results:**

Transcriptomic analysis revealed significant differential expression patterns between pre-diabetic and control groups. Four model combinations demonstrated superior predictive performance: Lasso+K-Nearest Neighbors, Elastic Net + K-Nearest Neighbors, Elastic Net + Random Forest, and Support Vector Machine+K-Nearest Neighbors. These models achieved high accuracy in predicting diabetes onset up to 46 months before clinical diagnosis. Both Elastic Net-based models achieved perfect classification performance in the validation cohort, demonstrating their potential as clinically viable diagnostic tools.

**Conclusion:**

This study establishes the feasibility of integrating peripheral blood transcriptomic profiling with machine learning for early pediatric T1DM prediction. The identified transcriptomic signatures and validated predictive models provide a foundation for developing clinically translatable, non-invasive diagnostic tools. These findings support the implementation of precision medicine approaches for childhood diabetes prevention and warrant validation in larger, multi-center cohorts to assess generalizability and clinical utility.

## Introduction

Diabetes mellitus, a chronic metabolic disorder characterized by elevated blood glucose levels, has become a major global health challenge (1). According to the International Diabetes Federation (IDF), over 537 million people worldwide were living with diabetes in 2021, a figure projected to increase significantly in the coming decades (2). Type 1 diabetes (T1D) and type 2 diabetes (T2D) represent the two primary forms of the disease, with T2D largely driven by lifestyle factors and T1D being an autoimmune condition typically diagnosed in childhood or adolescence (3, 4). The rising incidence of T1D in children has raised alarm, underscoring the urgent need for early intervention strategies (5). Children with diabetes, particularly those with T1D, face substantial long-term health risks, including cardiovascular disease, kidney failure, and neuropathy, making early detection and management critical to improving clinical outcomes and reducing long-term complications (6). Despite significant advances in diabetes care and treatment, early diagnosis remains a major challenge, as current methods often rely on clinical symptoms, which can appear after the disease has already progressed.

Early prediction of diabetes is crucial for several reasons, particularly in mitigating the long-term complications associated with the disease (7). Diabetes, particularly when diagnosed late, is often accompanied by irreversible damage to organs such as the heart, kidneys, and eyes (8). Early detection allows for timely interventions, including lifestyle modifications, pharmacological treatments, and regular monitoring, which can prevent or delay the onset of more severe complications. In the case of T1D, which often manifests during childhood, early diagnosis can enable better management of blood glucose levels, reducing the risk of diabetic ketoacidosis, a potentially life-threatening condition (9). Recent efforts in diabetes research have focused on identifying early biomarkers and developing diagnostic tools that can predict the onset of the disease before clinical symptoms emerge. Transcriptomics, the study of gene expression profiles, offers a promising avenue for identifying biomarkers that may reflect the early molecular changes associated with diabetes. In particular, profiling gene expression in peripheral blood has emerged as a non-invasive method for detecting alterations in gene expression patterns that may precede clinical diagnosis (10). Recent studies have demonstrated that changes in gene expression in prediabetic individuals, even in the absence of overt symptoms, can provide valuable insights into the underlying pathophysiology of diabetes. Furthermore, the application of machine learning (ML) algorithms to transcriptomic data has proven effective in enhancing the accuracy and precision of early diagnosis models (11). Several studies have employed ML models to predict the development of diabetes in high-risk populations, with promising results (12, 13). However, the majority of these studies have focused on adult populations, and much remains to be understood about how these approaches can be translated to pediatric populations, where the disease progression and risk factors may differ significantly.

**Figure 1:**
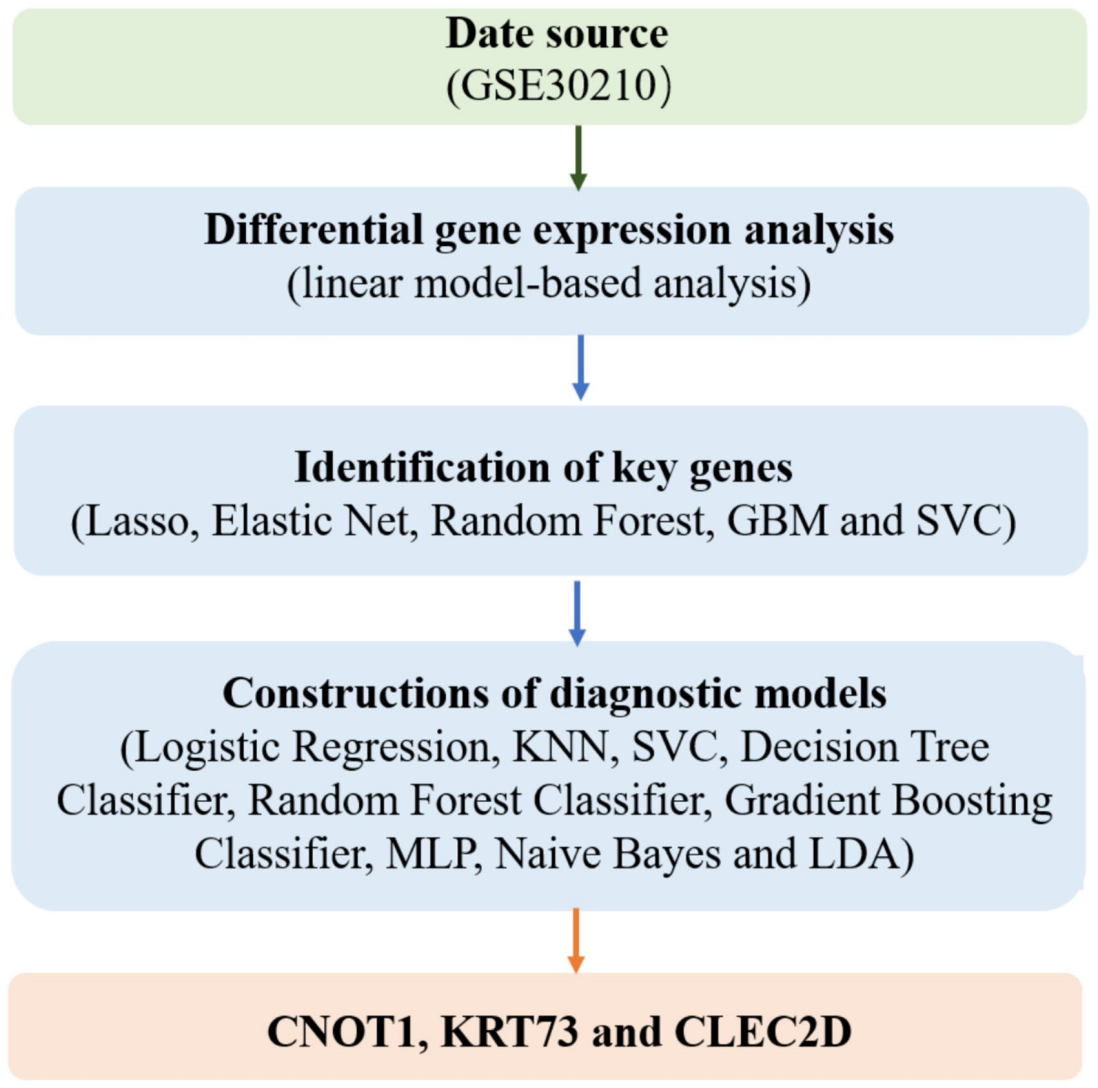
The scheme of research.

As shown in Figure 1, the present study aims to leverage advanced machine learning techniques and transcriptomic data to facilitate the early prediction of childhood diabetes, offering a novel and non-invasive approach to disease diagnosis. Our research focuses on analyzing peripheral blood RNA samples to identify gene expression patterns that can serve as potential biomarkers for diabetes prediction, well ahead of clinical diagnosis. By combining transcriptomic data with state-of-the-art machine learning algorithms, we aim to develop a robust diagnostic model capable of identifying prediabetic states in children as early as 46 months before the onset of clinical symptoms.

## Methods

### Data source

The transcriptomic data utilized in this study was derived from the public dataset GSE30210, which encompasses genome-wide expression profiling of children at risk of developing T1D.^1^ The dataset includes 247 peripheral blood RNA samples, collected from 18 prediabetic children and their matched controls, with the aim of uncovering the genes and molecular pathways involved in the early stages of T1D pathogenesis (Supplementary Table S1). Further details on these data can be found in the original publications (14, 15). The children in the study were selected based on their development of T1D-specific autoantibodies, a key early indicator of the disease (16). Each prediabetic child was matched with a persistently autoantibody-negative control child, ensuring similarity in terms of HLA-DQB1 risk category, gender, and geographic and temporal factors. The dataset was generated using Illumina Human HT-12 Expression BeadChips, a powerful tool for high-throughput gene expression analysis (17). This large-scale, longitudinal data provides a unique opportunity to study the gene expression changes that occur during the preclinical phase of T1D, offering a rich resource for identifying potential biomarkers for early diagnosis. The inclusion of matched controls allows for more accurate differentiation between disease-related gene expression patterns and normal biological variation, laying the foundation for machine learning-based approaches to predict the onset of diabetes in children well before clinical symptoms manifest.

### Differential gene expression analysis

For the differential gene expression analysis, the raw gene expression data obtained from the GSE30210 dataset were first pre-processed and normalized using the normalizeBetweenArrays function in the limma package to account for systematic biases across samples (18). Given that multiple peripheral blood RNA samples were collected longitudinally from each child, we implemented two complementary approaches to identify differentially expressed genes between prediabetic children and healthy controls. First, we accounted for the repeated measures design by estimating the intra-individual correlation using the duplicateCorrelation function. This approach models the correlation structure within each individual, analogous to including a random effect term, and provides a consensus correlation estimate shared across genes. A linear model was fitted using lmFit, incorporating the estimated correlation and block structure (individual IDs), followed by empirical Bayes moderation using eBayes. Second, for comparison, we performed a conventional linear model-based analysis without adjusting for intra-individual correlation. In this approach, lmFit and eBayes were applied directly using the same design matrix but without specifying a block structure. In both approaches, group information (prediabetic vs. control) was extracted from the phenotype data associated with the dataset and used to define the comparison groups. The resulting outputs provided sets of genes exhibiting statistically significant expression changes between the two groups, forming the basis for subsequent feature selection and machine learning modeling (19).

### Identification of key genes

For the identification of key genes, we utilized a machine learning-based feature selection approach. After preprocessing the gene expression data and defining the group labels (control and test), we applied several feature selection algorithms to refine the list of candidate genes. First, the data was split into training and test sets using an 80–20 ratio (20). We then implemented a range of machine learning algorithms for feature selection, including Lasso and ElasticNet, which are effective for handling high-dimensional data by performing both regularization and feature selection (21, 22). Additionally, Random Forest and Gradient Boosting Classifiers were employed for their ability to assess feature importance based on ensemble learning techniques (23). Support Vector Classifiers (SVC) were also used for their robustness in classification tasks (24). The SelectFromModel method from scikit-learn was applied to select the most important features based on the model’s output, identifying a set of genes that exhibited strong discriminatory power between prediabetic and control groups (25). This machine learning-based approach allowed us to narrow down the list of genes to those most likely to be associated with the onset of T1D. To explore the biological significance of the different subsets of genes identified, we performed functional enrichment analysis using Gene Ontology (GO) and Kyoto Encyclopedia of Genes and Genomes (KEGG) pathways. Genes were annotated and mapped to GO terms (biological process, molecular function, and cellular component) and KEGG pathways. Enrichment significance was evaluated using a hypergeometric test, with *p*-values adjusted for multiple comparisons using the Benjamini-Hochberg method. Enriched pathways and terms with adjusted *p*-values < 0.05 were considered statistically significant. The results provided insight into key molecular functions, biological processes, and pathways potentially involved in the early stages of type 1 diabetes development.

### Constructions of diagnostic models

To construct the diagnostic model for predicting childhood diabetes, a range of machine learning classifiers was employed to assess their performance in distinguishing between prediabetic children and healthy controls. After preprocessing and feature selection, we used a diverse set of algorithms to train models on the selected features. These algorithms included Logistic Regression, K-Nearest Neighbors (KNN), SVC, Decision Tree Classifier, Random Forest Classifier, Gradient Boosting Classifier, Multilayer Perceptron (MLP), Naive Bayes, and Linear Discriminant Analysis (LDA). The performance of each classifier was evaluated using four key metrics: Accuracy, Precision, Recall, and F1 Score, all of which provide valuable insight into the models’ ability to correctly identify both prediabetic and control groups (26). By comparing the performance of these various models, the most effective diagnostic model was selected.

To enhance the robustness and generalizability of the diagnostic models, we further adopted a five-fold cross-validation strategy. Instead of relying on a single random train-test split, the dataset was partitioned into five stratified subsets with preserved class distribution. Each model was trained and evaluated five times, with a different fold used as the validation set in each iteration while the remaining four served as the training data (27). This approach minimized potential bias from a particular data split and provided a more reliable estimate of model performance. For each combination of feature selection method and classification algorithm, the cross-validation procedure yielded fold-specific estimates of Accuracy, Precision, Recall, and F1 Score.

### Model validation using independent qPCR dataset

To validate the performance and clinical feasibility of the top-performing models, we conducted an independent small-scale experimental study using peripheral blood samples collected from six pediatric subjects—three healthy controls and three children clinically diagnosed with type 1 diabetes. Total RNA was extracted from whole blood using the Qiagen RNeasy Mini Kit following the manufacturer’s protocol. RNA quantity and purity were assessed using a NanoDrop spectrophotometer, and only samples with an A260/A280 ratio between 1.8 and 2.1 were used for downstream analysis. Complementary DNA (cDNA) was synthesized from 1 μg of total RNA using the High-Capacity cDNA Reverse Transcription Kit (Applied Biosystems). This study was approved by the Ethics Committee (Ethics Review Approval Number: YLZYYLL-2024—KY-004).

Quantitative real-time PCR (qPCR) was performed on an ABI 7500 Real-Time PCR System using SYBR Green Master Mix (Applied Biosystems). Each qPCR reaction was carried out in triplicate, with a total reaction volume of 20 μL containing 10 μL of SYBR Green Master Mix, 0.5 μL of each primer (10 μM), 2 μL of cDNA template, and nuclease-free water. The housekeeping gene GAPDH was used as an internal control. The Ct values for each target gene were normalized against GAPDH using the ΔCt method. To ensure compatibility with the machine learning models trained on normalized microarray data, the ΔCt values were then inverted (−ΔCt) and log2-transformed. The resulting expression matrix was used as input for the trained Elastic Net + K-Nearest Neighbors and Elastic Net + Random Forest models to assess classification performance on this external qPCR dataset.

### Software and tools

Differential expression analysis was performed using the limma package (version 3.62.2) in R 4.2.3. The identification of key genes and the construction of diagnostic models were implemented using sklearn in Python 3.12.

## Results

### Differential gene expression results

**Figure 2:**
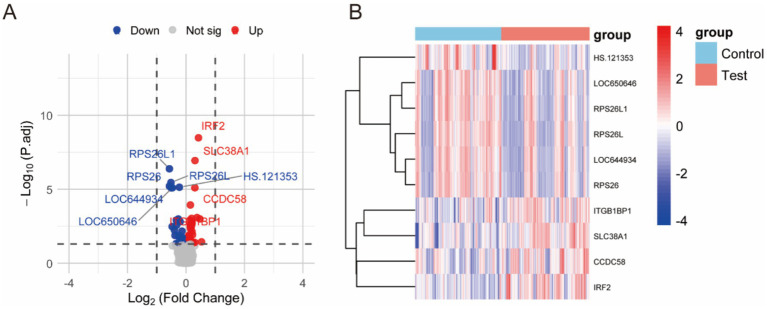
Differential gene expression between prediabetic children and healthy controls when intra-individual correlation was not accounted for. **(A)** The differences gene between two groups. **(B)** The heatmap of different groups.

When intra-individual correlation was not accounted for, as shown in Figure 2A, a total of 65 genes exhibited differential expression between prediabetic children and healthy controls. The top ten differentially expressed genes include: IRF2, SLC38A1, RPS26L1, RPS26L, RPS26, HS.121353, CCDC58, LOC644934, LOC650646, and ITGB1BP1 (Figure 2B; Table 1). When intra-individual correlation was accounted for, a total of 37 genes exhibited differential expression between prediabetic children and healthy controls. The top ten differentially expressed genes include: IRF2, SLC38A1, RPS26L1, RPS26L, HS.121353, RPS26, CCDC58, LOC644934, LOC650646, ITGB1BP1 (Supplementary Table S2).

**Table 1:** The top ten differentially expressed genes.

ILMN_Gene	B	logFC	AveExpr	adj. P. Val
IRF2	20.89	0.43	9.94	<0.001
SLC38A1	16.88	0.31	9.34	<0.001
RPS26L1	15.01	−0.57	10.47	<0.001
RPS26L	12.77	−0.51	10.66	<0.001
RPS26	12.08	−0.57	11.98	<0.001
HS.121353	11.77	−0.23	6.92	<0.001
CCDC58	11.35	0.30	7.47	<0.001
LOC644934	11.43	−0.51	10.53	<0.001
LOC650646	11.50	−0.46	9.14	<0.001
ITGB1BP1	8.66	0.15	7.52	<0.001

### Identification of key genes

The machine learning-based feature selection process identified a set of key genes that consistently appeared across multiple models, highlighting their potential relevance in the prediction of childhood diabetes. Using five different feature selection techniques—Lasso, Elastic Net, Random Forest, Support Vector Machine (SVM), and Gradient Boosting Machine (GBM)—several genes were identified as important markers for differentiating prediabetic children from healthy controls. When intra-individual correlation was not accounted for, feature selection using the five machine learning models identified a subset of 18–40 key genes (Table 2). Notably, CNOT1, KRT73, and CLEC2D were selected by multiple algorithms, underscoring their potential as biomarkers. Other genes, such as GCC2, CCDC58, and ITGB1BP1, were also identified as significant across different models, suggesting their possible involvement in the molecular pathways leading to T1D. The consistency across different classifiers further strengthens the reliability of these genes as potential diagnostic biomarkers. The results highlight a robust set of genes that warrant further investigation to validate their role in early diabetes prediction and to explore their mechanistic implications in disease progression. When intra-individual correlation was accounted for, feature selection using the five machine learning models identified a subset of 14–21 key genes (Supplementary Table S3). The results of functional enrichment analysis for all gene sets can be found in Supplementary Table S4.

**Table 2:** A subset of key genes identified by five machine learning models when intra-individual correlation was not accounted for.

Lasso	Elastic Net	Random Forest	SVM	GBM
CNOT1	CNOT1	IKZF1	CNOT1	KRT73
KRT73	KRT73	LOC644934	KRT73	CLEC2D
CLEC2D	CLEC2D	CLEC2D	CLEC2D	CCDC58
GCC2	GCC2	GCC2	GCC2	RPS26L
THEM4	THEM4	CCDC58	THEM4	KRT72
CCDC58	CCDC58	ITGB1BP1	CCDC58	LOC441763
ITGB1BP1	ITGB1BP1	RPS26L	KRT72	MT1F
LOC387820	LOC387820	MYOM2	GDPD5	FOLR3
KRT72	KRT72	CCT8	FOLR3	P2RX1
LSM11	SH3PXD2A	LOC650646	TFB1M	IRF2
LOC441763	HIST1H2BD	FOLR3	PHACTR4	SLC38A1
CCT8	IRF2	SLC11A1	SH3PXD2A	XAB2
MT1F	SLC38A1	P2RX1	LOC645899	RBPMS2
SH3PXD2A	BTNL3	IRF2	P2RX1	HS.373705
LOC645899	PNMA3	SLC38A1	HIST1H2BD	HS.121353
P2RX1	LOC648226	XAB2	IRF2	ZNF595
HIST1H2BD	AOF2	LOC648226	ABCA1	EEF1G
IRF2	HS.447508	HS.46689	BTNL3	FOXK2
SLC38A1	ZNF595	HS.121353	CPA5	
XAB2	MT1E	ZNF595	LOC648226	
BTNL3	RPS26L1	C10ORF32	HS.196073	
PNMA3	RPS26	RPS26L1	HS.571875	
CPA5	EEF1G	EEF1G	HS.153034	
LOC648226	FOXK2	IRF5	HS.571151	
RBPMS2			HS.121353	
AOF2			ZNF595	
HS.196073			AQP10	
HS.571875			MT1E	
HS.153034			EEF1G	
HS.571151			FOXK2	
HS.447508				
HS.121353				
ZNF595				
MT1E				
RPS26L1				
RPS26				
EEF1G				
FOXK2				
IRF5				
WAC				

### Machine learning diagnostic model results

The machine learning diagnostic models constructed using the selected gene features were evaluated on their ability to distinguish between prediabetic children and healthy controls. A total of nine machine learning algorithms—Logistic Regression, KNN, SVC, Decision Tree Classifier, Random Forest Classifier, GBM, MLP, Naive Bayes, and LDA—were applied to the selected gene set. The models were trained using the gene expression data, which had undergone feature selection to retain only the most discriminative genes identified through Lasso, Elastic Net, Random Forest, SVM, and GBM techniques. The performance of the diagnostic models was evaluated using four key metrics: accuracy, precision, recall, and F1 score. These metrics were calculated for a range of machine learning model combinations, each utilizing a different set of feature selection techniques and classifiers. A total of 45 unique combinations were tested, resulting in consistently high performance across multiple models.

When intra-individual correlation was not accounted for, as show in Figure 2, among the best-performing models, Lasso + KNN, Elastic Net + KNN, and Elastic Net + Random Forest achieved perfect classification performance, with all models yielding an accuracy of 1.0, precision of 1.0, recall of 1.0, and an F1 score of 1.0 (Table 3). These results indicate that these models successfully classified all test instances with no false positives or false negatives, suggesting excellent generalization to new data. Similarly, combinations like SVM + KNN also achieved perfect performance metrics, demonstrating that a variety of model pairings with specific feature sets can provide robust predictive capabilities. Other combinations, such as Lasso + Logistic Regression, Elastic Net + Logistic Regression, Elastic Net + Multilayer Perceptron, and Random Forest + KNN, all produced very high performance, with accuracy of 0.98, precision of 1.0, recall of approximately 0.96, and F1 score of around 0.98. These results suggest that while these models did not achieve perfect classification (with a slight decrease in recall), their overall predictive ability remained exceptionally high. The slight drop in recall, which measures the proportion of true positives identified, could be indicative of the models’ slightly more conservative classification of the positive class, which may help reduce false positives but slightly increase false negatives. When intra-individual correlation was accounted for, the best-performing models were Random Forest + K-Nearest Neighbors, Random Forest + Support Vector Machine, and SVM + Multilayer Perceptron, each achieving an accuracy of 0.98 (Supplementary Table S5). Since the models generated without accounting for intra-individual correlation demonstrated higher overall accuracy, we selected the results from the analysis without intra-individual correlation for further evaluation.

**Table 3:** The classification performance results of machine learning diagnostic model.

Feature selection method	Classifier	Accuracy	Precision	Recall	F1 score
Lasso	K-Nearest Neighbors	1	1	1	1
Elastic Net	K-Nearest Neighbors	1	1	1	1
Elastic Net	Random Forest	1	1	1	1
SVM	K-Nearest Neighbors	1	1	1	1
Lasso	Logistic Regression	0.98	1	0.958	0.979
Elastic Net	Logistic Regression	0.98	1	0.958	0.979
Elastic Net	Multilayer Perceptron	0.98	1	0.958	0.979
Random Forest	K-Nearest Neighbors	0.98	1	0.958	0.979
SVM	Logistic Regression	0.98	1	0.958	0.979
SVM	Support Vector Machine	0.98	1	0.958	0.979
SVM	Random Forest	0.98	1	0.958	0.979
SVM	Multilayer Perceptron	0.98	1	0.958	0.979
SVM	Linear Discriminant Analysis	0.98	1	0.958	0.979
Lasso	Support Vector Machine	0.96	0.958	0.958	0.958
Lasso	Naive Bayes	0.96	1	0.917	0.957
Elastic Net	Support Vector Machine	0.96	0.958	0.958	0.958
Elastic Net	Linear Discriminant Analysis	0.96	0.958	0.958	0.958
Random Forest	Random Forest	0.96	1	0.917	0.957
SVM	Gradient Boosting Machine	0.96	1	0.917	0.957
Lasso	Random Forest	0.94	0.920	0.958	0.939
Lasso	Gradient Boosting Machine	0.94	0.957	0.917	0.936
Lasso	Multilayer Perceptron	0.94	0.920	0.958	0.939
Lasso	Linear Discriminant Analysis	0.94	0.920	0.958	0.939
Elastic Net	Gradient Boosting Machine	0.94	0.920	0.958	0.939
Random Forest	Gradient Boosting Machine	0.94	1	0.875	0.933
GBM	Random Forest	0.94	1	0.875	0.933
GBM	Multilayer Perceptron	0.94	0.957	0.917	0.936
Random Forest	Support Vector Machine	0.92	0.917	0.917	0.917
GBM	K-Nearest Neighbors	0.92	0.955	0.875	0.913
GBM	Support Vector Machine	0.92	0.917	0.917	0.917
Elastic Net	Naive Bayes	0.9	0.880	0.917	0.898
Random Forest	Logistic Regression	0.9	0.913	0.875	0.894
Random Forest	Decision Tree	0.9	0.913	0.875	0.894
Random Forest	Multilayer Perceptron	0.9	0.880	0.917	0.898
Random Forest	Linear Discriminant Analysis	0.9	0.913	0.875	0.894
SVM	Naive Bayes	0.9	0.913	0.875	0.894
GBM	Logistic Regression	0.9	0.913	0.875	0.894
GBM	Gradient Boosting Machine	0.9	0.952	0.833	0.889
GBM	Naive Bayes	0.9	0.952	0.833	0.889
GBM	Linear Discriminant Analysis	0.9	0.913	0.875	0.894
Random Forest	Naive Bayes	0.88	0.875	0.875	0.875
Elastic Net	Decision Tree	0.8	0.769	0.833	0.800
SVM	Decision Tree	0.8	0.818	0.750	0.783
GBM	Decision Tree	0.78	0.933	0.583	0.718
Lasso	Decision Tree	0.74	0.720	0.750	0.735

**Figure 3:**
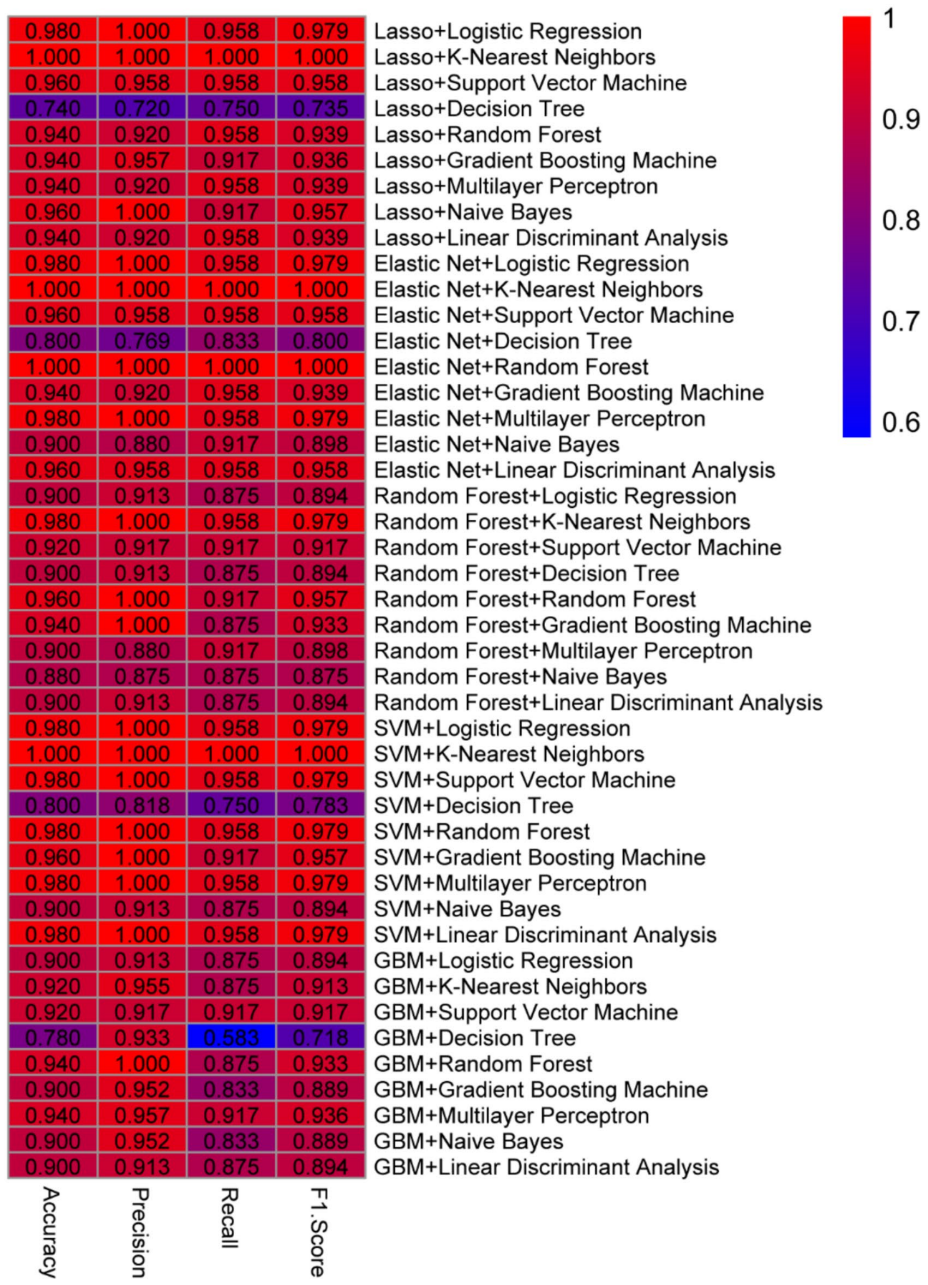
Different model combinations evaluated.

To validate the stability and generalizability of the diagnostic models, five-fold cross-validation was conducted on each combination of feature selection method and classifier. As shown in Supplementary Table S6, the models combining Lasso + K-Nearest Neighbors (KNN), Elastic Net + KNN, and Elastic Net + Random Forest continued to demonstrate strong predictive performance across all five folds. The Lasso + KNN model achieved an average accuracy of 0.9796, with precision, recall, and F1 scores consistently above 0.93. Notably, this model reached perfect classification (accuracy = 1.0, precision = 1.0, recall = 1.0, F1 = 1.0) in three out of five folds, while maintaining high recall (0.88–0.92) in the remaining folds. Similarly, the Elastic Net + KNN model exhibited strong but slightly more variable performance, with accuracy ranging from 0.898 to 1.0 and an average F1 score of 0.953. Despite a slight dip in recall in one fold (0.80), this model still maintained excellent precision throughout all iterations. The Elastic Net + Random Forest model also performed robustly, achieving perfect classification in two out of five folds and maintaining high scores across the board (average accuracy = 0.955, average F1 score = 0.956). Notably, this model yielded recall values of 0.88 or higher in all folds, indicating reliable sensitivity in identifying prediabetic cases. Overall, these cross-validated results reaffirm the effectiveness of Lasso and Elastic Net-based feature selection methods in combination with KNN and Random Forest classifiers. The consistent high performance across multiple folds highlights their potential utility in the reliable early diagnosis of childhood diabetes (Figure 3).

### Selected biomarkers in different classification techniques

**Figure 4:**
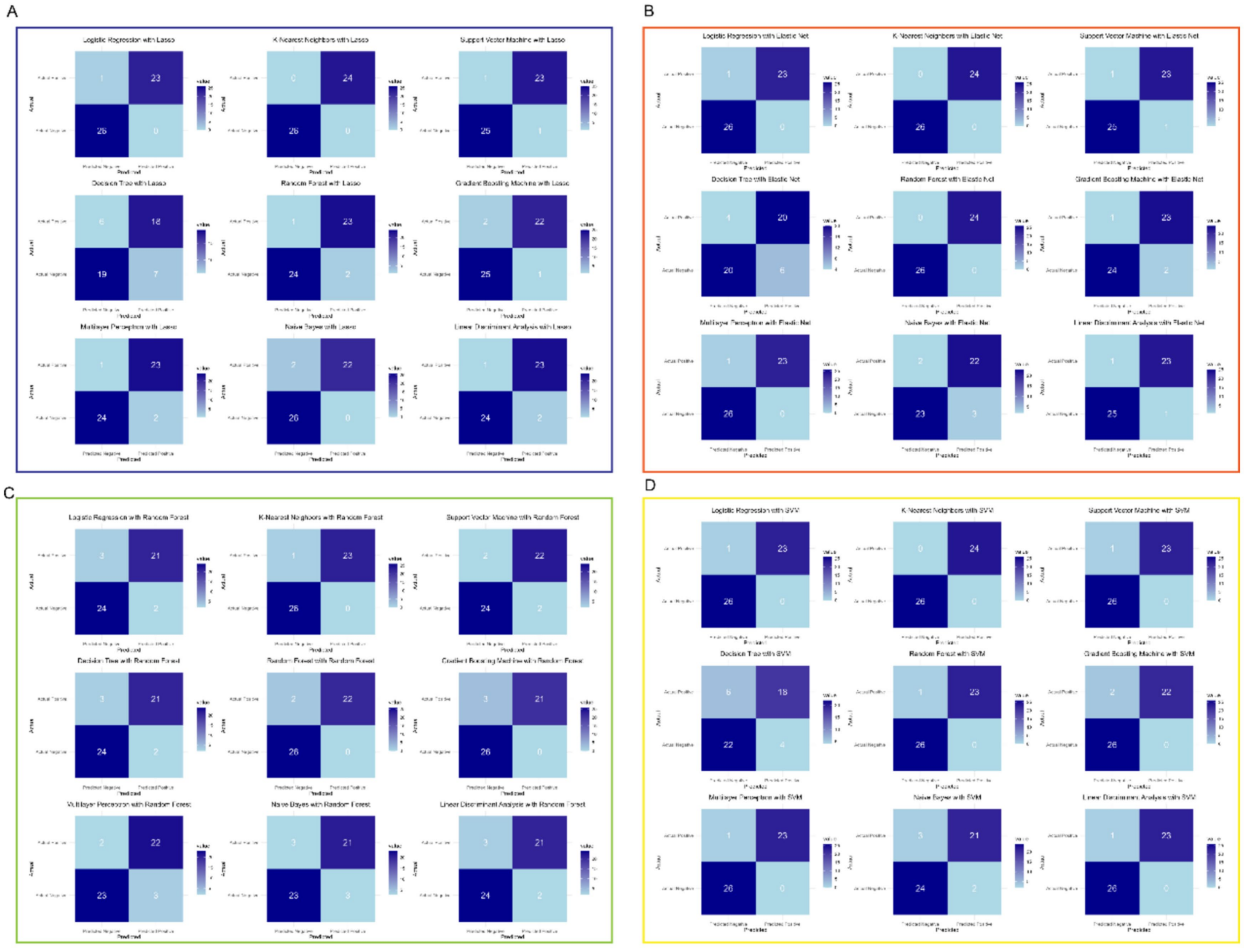
Selected feature set derived from machine learning-based feature selection. **(A)** Lasso with multiple models. **(B)** Elastic Net with multiple models. **(C)** Random Forest with multiple models. **(D)** SVM with multiple models.

As show in Figure 4, the consistent high performance across multiple models indicates that the selected feature set, derived from the machine learning-based feature selection, is highly discriminative and robust for distinguishing between prediabetic children and healthy controls. Importantly, the use of a diverse set of machine learning algorithms—including linear models, ensemble methods, and non-linear classifiers—demonstrates the versatility and reliability of the selected biomarkers across different classification techniques. Furthermore, the comparison of models shows that KNN, when combined with Lasso or Elastic Net for feature selection, tends to provide optimal results, suggesting that this classifier is particularly well-suited to the problem of early diabetes prediction based on gene expression data. The high performance of SVM and Random Forest models further supports the effectiveness of these algorithms for high-dimensional biomedical data classification tasks. Overall, these results underscore the potential of combining advanced feature selection techniques with machine learning classifiers to create highly accurate and reliable models for the early prediction of childhood diabetes, highlighting a promising avenue for the development of diagnostic tools in clinical practice.

### Model validation

**Figure 5:**
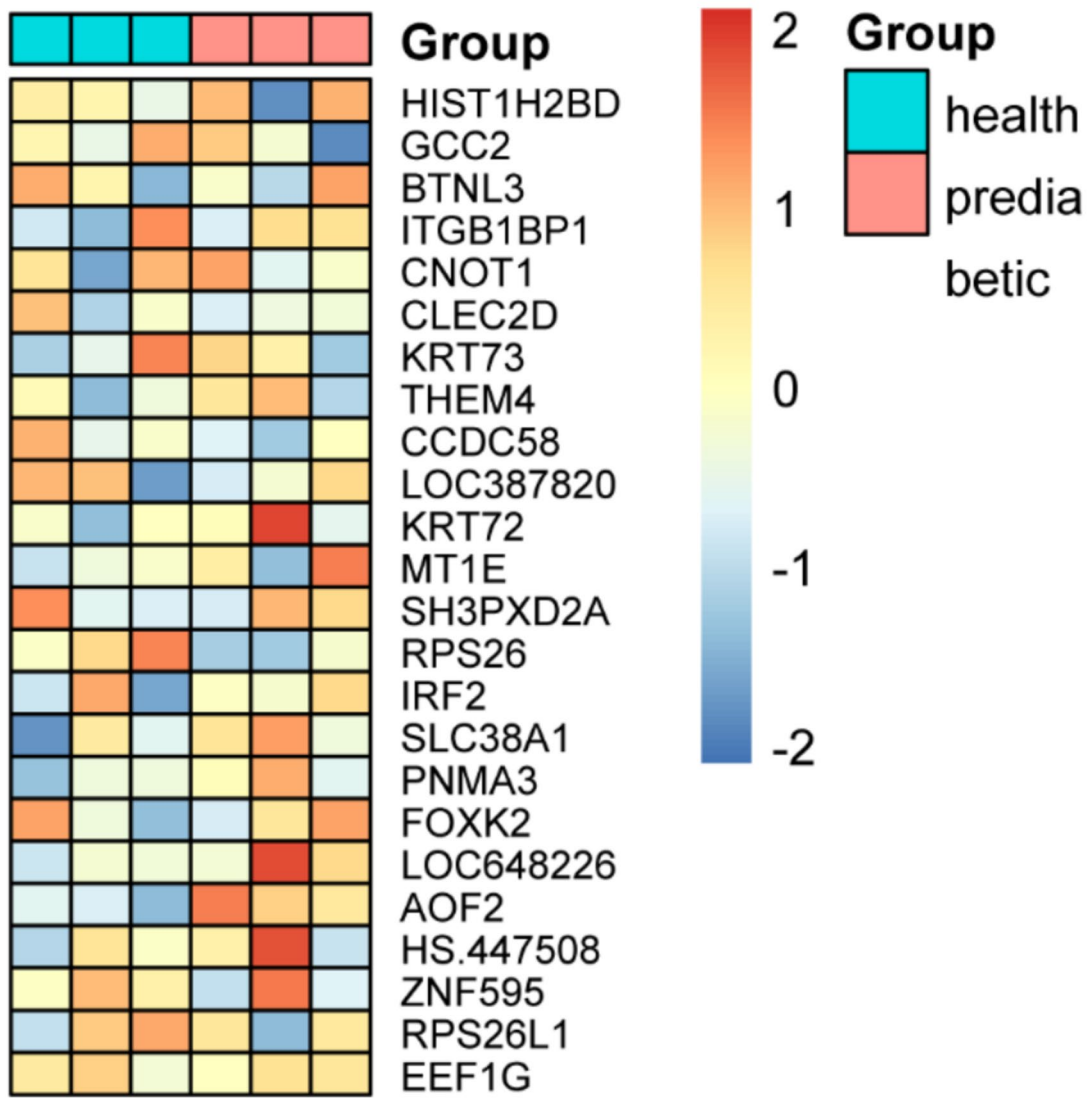
Expression profiles of 24 key genes and classification performance validation using qPCR-based independent dataset.

The expression levels of the 24 key genes in the six clinical samples are shown in Figure 5. In the independent validation using the qPCR-based dataset, both the Elastic Net + K-Nearest Neighbors and Elastic Net + Random Forest models successfully classified all six samples correctly, achieving 100% accuracy. Notably, these two models required the fewest input genes (*n* = 24) among the top-performing combinations, underscoring their practicality for clinical implementation. This real-world validation reinforces the robustness and generalizability of the selected models and supports their potential use in early diagnostic workflows for childhood diabetes.

## Discussion

Our results of this study demonstrate a robust and highly discriminative set of differentially expressed genes that effectively distinguish prediabetic children from healthy controls. Through the analysis involving differential gene expression and machine learning-based feature selection, we identified a subset of key genes, including CNOT1, KRT73, and CLEC2D, which consistently emerged across multiple models as potential biomarkers for early diabetes prediction. The application of nine machine learning algorithms, combined with five feature selection techniques, yielded diagnostic models with exceptional performance, particularly when using Lasso or Elastic Net in conjunction with KNN, which achieved perfect classification metrics (accuracy, precision, recall, and F1 score of 1.0). These findings highlight the reliability and versatility of the selected gene set across diverse classification approaches, underscoring their potential utility in developing accurate diagnostic tools for childhood diabetes. The high performance of these models, coupled with the consistency of key gene identification, provides a strong foundation for further validation and exploration of their mechanistic roles in disease progression.

The exceptional performance of the machine learning models, particularly those combining Lasso or Elastic Net with KNN, underscores the robustness of the selected gene features in capturing the molecular signatures associated with prediabetes in children. The perfect classification metrics (accuracy, precision, recall, and F1 score of 1.0) achieved by these models suggest that the identified gene set is not only highly discriminative but also generalizable, with minimal risk of overfitting. This is particularly noteworthy given the complexity and high dimensionality of gene expression data, where the risk of model overfitting is often a concern. The consistency of key genes, such as CNOT1, KRT73, and CLEC2D, across multiple feature selection methods and classifiers further strengthens their candidacy as reliable biomarkers. These genes may play critical roles in the molecular pathways underlying early diabetes development, potentially involving immune regulation, cellular stress responses, or metabolic dysregulation. For instance, CNOT1, a component of the CCR4-NOT complex, is known to regulate mRNA stability and translation, processes that could be dysregulated in metabolic disorders (28, 29). Similarly, CLEC2D, a C-type lectin domain family member, has been implicated in immune modulation, suggesting a possible link to the autoimmune processes often observed in T1D (30). The inclusion of genes like GCC2 and ITGB1BP1, which are involved in intracellular trafficking and cell adhesion, respectively, further hints at the multifaceted nature of the disease, involving both metabolic and structural cellular changes (31, 32).

Moreover, the high performance of models like SVM and Random Forest, which are well-suited for handling high-dimensional data, highlights the adaptability of these algorithms to complex biomedical datasets. The slight variation in recall observed in some models, such as Lasso + Logistic Regression, may reflect a trade-off between sensitivity and specificity, which could be further optimized depending on clinical priorities. For example, in a diagnostic setting, minimizing false negatives (high recall) might be prioritized to ensure early intervention, even at the cost of slightly increased false positives. The success of KNN in this context is particularly intriguing, as its non-parametric nature allows it to capture subtle patterns in the data without imposing strong assumptions, making it an ideal choice for gene expression analysis where the underlying data distribution may not be well-defined (33).

The final 24-gene signature provides important insights into the molecular mechanisms underlying type 1 diabetes pathogenesis and highlights potential avenues for biomarker discovery and therapeutic intervention. Several genes in the signature are directly implicated in immune regulation and inflammation, which are central to the autoimmune destruction of pancreatic β-cells characteristic of T1D (34, 35). For instance, IRF2 (Interferon Regulatory Factor 2) plays a critical role in modulating interferon signaling and immune responses, and its dysregulation has been associated with autoimmune diseases (36). CLEC2D, encoding a C-type lectin domain family member, participates in natural killer (NK) cell-mediated cytotoxicity, potentially contributing to β-cell destruction (37). SH3PXD2A and ITGB1BP1 are involved in cytoskeletal remodeling and cell adhesion, processes that may influence immune cell infiltration and islet architecture integrity during T1D development (38, 39). Metabolic and stress response pathways are also represented within the signature. SLC38A1, a sodium-coupled neutral amino acid transporter, may reflect altered nutrient sensing or metabolic stress in immune or pancreatic cells (40). THEM4, implicated in mitochondrial function and apoptosis regulation, could contribute to β-cell vulnerability under autoimmune attack (41). Furthermore, MT1E, a metallothionein, and ZNF595, a zinc finger protein, are linked to cellular stress responses and transcriptional regulation, potentially modulating β-cell survival during disease progression (42, 43). Epigenetic and transcriptional regulators within the gene set, including CNOT1 (a key component of the CCR4-NOT transcription complex), FOXK2, and HIST1H2BD, suggest that transcriptional and chromatin remodeling processes are integral to early T1D molecular alterations (28, 44). Dysregulation of these genes may affect gene networks controlling immune tolerance, β-cell identity, or apoptosis. Ribosomal and translational machinery components, such as RPS26, RPS26L1, and EEF1G, highlight potential shifts in protein synthesis capacity or cellular homeostasis that accompany preclinical diabetes (45, 46). Importantly, several of these genes (e.g., IRF2, SLC38A1, CLEC2D) have been previously implicated as potential biomarkers or functional mediators in autoimmune or metabolic disorders, supporting their relevance for early detection strategies. The integration of genes involved in immune modulation, metabolism, transcriptional regulation, and cellular stress underscores the multi-faceted nature of T1D pathogenesis and identifies promising candidates for further mechanistic studies and therapeutic targeting. These findings provide a biologically coherent basis for the diagnostic model and reinforce the translational potential of the identified gene set for precision medicine approaches in pediatric diabetes.

Moreover, it is important to consider how age-dependent transcriptomic variation and immune ontogeny may influence the observed gene expression patterns and their diagnostic relevance in pediatric populations. The human immune system undergoes significant maturation during childhood, involving dynamic shifts in innate and adaptive immunity, lymphocyte repertoires, and cytokine responses (47). These developmental processes can impact baseline and stimulus-induced gene expression, potentially altering the biomarker landscape across age groups. For example, genes involved in immune regulation, such as IRF2 and CLEC2D, may exhibit distinct expression kinetics during early life, reflecting evolving immunological competence (48, 49). Additionally, epigenetic regulation and chromatin remodeling, mediated by factors like CNOT1 and FOXK2, are known to be modulated by developmental stage and environmental exposures, further influencing transcriptomic signatures in children (50, 51). Thus, the diagnostic utility of the identified gene set may be uniquely optimized for the pediatric window, underscoring the importance of age-specific biomarker validation and the integration of developmental immunology into future study designs.

In biomedical machine learning studies, especially those involving small sample sizes, the use of robust validation strategies is critical to ensure model reliability and generalizability (10). Simple train-test splits may lead to overfitting or overly optimistic performance estimates. Therefore, we employed five-fold cross-validation to mitigate sampling bias and assess the consistency of model performance across different data partitions. This approach provides a more realistic evaluation of the model’ s predictive ability and reduces the likelihood of false-positive findings. Future studies should also consider alternative methods such as bootstrapping or external validation to further confirm the robustness of diagnostic models.

Several limitations should be acknowledged. First, the sample size, though sufficient for initial discovery, may limit the generalizability of the findings to broader populations. Larger, multi-center cohorts are needed to validate the robustness of the identified biomarkers and ensure their applicability across diverse demographic and genetic backgrounds. The exceptionally high classification metrics observed in our study may reflect potential overfitting, particularly given the limited sample size and the use of a single train-test split for model evaluation. While the external validation provided some support for the robustness of our models, future work should include larger, multi-center cohorts and independent replication datasets to further assess generalizability. Second, the study focused solely on gene expression data, which, while informative, does not capture the full complexity of diabetes pathogenesis. Integrating additional omics data, such as proteomics, metabolomics, and epigenetics, could provide a more comprehensive understanding of the molecular mechanisms underlying prediabetes and improve the predictive power of the models, as demonstrated by recent studies employing multiomics and explainable artificial intelligence approaches for early diagnosis of insulin resistance and related metabolic conditions (52). Third, the machine learning models, though highly accurate, were trained and tested on the same dataset, which may introduce bias. External validation using independent datasets is essential to confirm the models’ performance and generalizability. Additionally, while the selected genes show strong potential as biomarkers, their functional roles in diabetes development remain to be elucidated. Further experimental studies are needed to explore the biological pathways involving these genes and their contribution to disease progression. Lastly, the clinical translation of these findings requires careful consideration of practical challenges, such as the cost and feasibility of implementing gene expression profiling in routine clinical practice. Addressing these limitations in future research will be critical for advancing the development of reliable diagnostic tools and improving early intervention strategies for childhood diabetes.

In addition to addressing practical challenges such as cost and feasibility, future clinical translation of our findings will also depend on the use of predictive models that are interpretable and understandable by clinical professionals. While our selected models—K-Nearest Neighbors and Random Forest—demonstrated high predictive accuracy, they are not inherently interpretable, which may hinder their acceptance and utility in real-world clinical settings. Therefore, integrating eXplainable Artificial Intelligence (XAI) techniques in future work is essential to enhance model transparency and foster trust among healthcare providers. XAI approaches can provide human-interpretable insights into model decision-making processes, facilitating responsible AI deployment in medicine (53, 54). Such strategies would support collaboration between human experts and AI systems, enabling more informed and ethical clinical decision-making in the context of early childhood diabetes diagnosis.

Overall, these findings not only validate the potential of machine learning-driven approaches for early diabetes prediction but also provide a framework for identifying and prioritizing key biomarkers for further mechanistic and clinical validation. The integration of advanced computational techniques with biological insights offers a powerful strategy for unraveling the complex etiology of childhood diabetes and paves the way for the development of precision diagnostic tools in clinical practice. Future studies should focus on validating these biomarkers in larger, independent cohorts and exploring their functional roles in disease progression, which could ultimately lead to targeted interventions and improved outcomes for at-risk children.

## Data Availability

The original contributions presented in the study are included in the article/Supplementary material, further inquiries can be directed to the corresponding authors.

## Footnotes

^1^ https://www.ncbi.nlm.nih.gov/geo/query/acc.cgi?acc=GSE30210
